# Coping strategies in parents of children with disabilities: A case‐control study

**DOI:** 10.1002/brb3.2701

**Published:** 2022-07-14

**Authors:** Francisco J. Alós, Andrés García García, Miguel A. Maldonado

**Affiliations:** ^1^ Psychological Care Unit Department of Psychology University of Córdoba Córdoba Spain; ^2^ Maimónedes Institute of Biomedical Research of Córdoba (IMIBIC), Reina Sofía University Hospital of Córdoba Córdoba Spain; ^3^ Department of Experimental Psychology University of Seville Seville Spain

**Keywords:** autism spectrum disorder, case‐control study, coping strategies, disability, parents, stressors

## Abstract

**Introduction:**

The aim of this article is to determine whether there are differences in the coping strategies of parents of children with disabilities (autism spectrum disorder or other disabilities) and children without disabilities, in reference to the most stressful situation they have experienced with their child in the last year.

**Method:**

To conduct the study, a purposive sample selection based on case‐control characteristics was carried out, in which a total sample of 170 participants was recruited. Participants were assigned, according to their characteristics, to the group of parents of children without disabilities, with ASD or with other disabilities. An ad hoc sociodemographic questionnaire and the Coping Responses Inventory for adults were administered.

**Results:**

The results obtained indicate that there are differences in the use of coping strategies between parents of children without disabilities and with disabilities but not between the two disability groups. Parents of children with disabilities have significantly higher scores on the four subscales defined as avoidance strategies, and on one subscale identified as an approach strategy.

**Conclusions:**

Parents of children with disabilities use avoidance strategies, to a greater extent, during the most stressful situations they have experienced in the last year with their child. In addition, they tend to use avoidance strategies regardless of the type of disability their child has.

## Graphical abstract

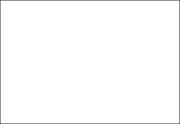

1

One of the main events that modify the family structure is the birth of a child. Parents restructure their functioning, creating expectations for the new family member, specifically, for the parents of children with disabilities, by altering expectations and causing conflicts or psychological imbalances (López, 2011).

In the field of psychology, stress has been investigated as the central factor related to the conflicts that people experience. According to Lazarus (1966), stress is understood as a term that encompasses problems that include the provocative stimulus, reactions of the human body and their intervention processes. Thus, the stress response would be a result of the interaction of individuals characteristics and environmental demands. There are a few studies that specifically examine how parents, who are responsible for children with disabilities, experience problems related to stress. The studies indicate that these parents also show higher levels of stress than the parents of children without disabilities (Baker‐Ericzén et al., 2005; Tomanik et al., 2004). In addition, specifically, parents of children with Autism Spectrum Disorder (ASD) experience higher levels of stress than parents of children with other types of disabilities (see Baker et al., 2005; Baker et al., 2003; Kasari & Sigman, 1997; Wolf et al., 1989). In a meta‐analysis by Lindo et al. (2016), it is pointed out that there are four types of stressors faced by parents of children with disabilities: relational (Freedman et al., 2012), financial (Parish et al., 2012), relatives of the family (Manor‐Binyamini & Abu‐Ajaj, 2012), and the decreased parenting efficacy (Karst & Van Hecke, 2012). Such challenges conduce higher levels of stress (Baker‐Ericzén et al., 2005; Benson, 2006; Brookman‐Frazee & Koegel, 2004; Minjarez et al., 2013; Tomanik et al., 2004) and can have a negative impact on the psychological well‐being of the parents (Trute & Hiebert‐Murphy, 2002).

A significant number of authors consider the management of stressors to be dependent on the type of stress that the family have faced (Berszan, 2017; Brands et al., 2018; Cantwell‐Barti, 2018; Dyson, 2010; Finset & Andersson, 2000; Paster et al., 2009; Tomberg et al., 2005; Twoy et al., 2007). According to Fleishman (1984), coping can be defined as the cognitive or behavioral responses that reduce or eliminate stress or both. According to this author, there are two types of coping strategies: approach and avoidance. Approaching the problem as a coping strategy reflects the cognitive and behavioral strength an individual has to manage or resolve stressful conditions. On the contrary, coping by avoidance shows the cognitive and behavioral attempts to avoid thoughts related to the stressors (Fleishman, 1984; Moos, 1988). In the model of coping by Moos (1985), it is pointed out that the characteristics of the stressor and the evaluation that the subject makes of the situation are predictive factors of the coping responses.

In addressing stressors, two complementary concepts related to coping need to be considered: styles and strategies. Coping styles are the conscious way to approach stress, while coping strategies refer to more specific actions, with the second concept as the more variable and with greater predictive capacity (Pelechano, 2000). The latter is also an adaptive factor that can help during high periods of stress (Lazarus & Folkman, 1984; Moos & Schaefer, 1993).

Two types of coping strategies can be distinguished: active and passive. Active coping strategies, such as approaching the problem, are more positive and resolutive for the family, while passive coping strategies, such as avoiding the problem, can be a mechanism to reduce stress short‐term. However, avoiding the problem rather than directly addressing the stressful events could be harmful (Benson, 2006; Dyson, 2010; Twoy et al., 2007).

As it points out in the last revision on styles of coping in families of children with disabilities, research is still limited (Isa et al., 2017). The few studies that have been made in this field have investigated the moderator effect of coping strategies and have found evidence that coping strategies by approach have a positive influence on psychological well‐being and the reduction of stress. Investigations made in this line of study point out that parents of children with disabilities, in general, employ passive strategies and avoid stressful situations, even though they use seeking social support as the only approach strategy (Paster et al., 2009; Twoy et al., 2007).  The methodology that has been used in this field of study has mainly been qualitative, with semistructured interviews, nonvalidated questionnaires and samples that range from 11 and 70 subjects (see Berszan, 2017; Brands et al., 2018; Cantwell‐Barti, 2018; Finset & Andersson, 2000; Paster et al., 2009; Tomberg et al., 2005; Twoy et al., 2007). Said studies have pointed out that parents of children with disabilities use more avoidance strategies than parents who do not have children with disabilities. However, given the limitations observed in these studies, it is necessary to reexamine this subject with more support: methodological and statistical.

The objective of this article is to investigate, coping strategies of the parents of children with and without disabilities given the most stressful situation they experienced with their child in the last year. In study, different coping strategies are analyzed between parents depending on the absence or presence of disability of their children (ASD, other disabilities and without disability).

The following hypotheses are derived from this objective:

*H0_1_*: There are no differences in the use of coping strategies between parents of children without disability and with disability (ASD and other disabilities).

*H1*: There are differences in the use of coping strategies between parents of children without disabilities and with disabilities (ASD and other disabilities).

*H0_2_*: There are no differences in the use of coping strategies between parents of children with ASD and other disabilities.

*H2*: There are differences in the use of coping strategies between parents of children with ASD and other disabilities.

## 2 METHOD

### 2.1 Participants

The sample consisted of 140 parents through intentional selection based on case and control characteristics: 70 parents of children with disabilities (physical disability, such as muscular dystrophy, or intellectual disability, such as Down syndrome, cerebral paralysis, or ASD) and 70 parents of children without disabilities (normalized development). Regarding the gender of the parents, 75.7% were female (*n* = 106) and 24.3% were male (*n* = 34). To access this sample according to the relevant characteristics (intentional selection) for the study (disability or nondisability), it was selected through regular schools (for children without disabilities) and associations (for children with disabilities), although eventually, the diagnosis of ASD, other disability, or no disability was self‐reported by the parents included in the study. The selected centers were located in a Spanish city, so it was necessary to adapt the questionnaire to Spanish. Participants were assigned to one of three groups according to the following clustering criteria: (1) type of disability of the children (ASD or other disabilities) and (2) disability or nondisability of the children. In this way, group 1 (G1) (without disability), group 2 (G2) (ASD), and group 3 (G3) (other disabilities) were formed (*M* = 1.7, *SD* = .78), as a result, 70 parents of children without disabilities (group G1) and 70 parents of children with disabilities (42 from group G2 and 28 from group G3), which together made up the 140 participants of the total sample. Therefore, an attempt was made to balance the number of participants with disability and without disability in order to be able to make the subsequent comparisons in the most appropriate way possible. For participation, the following admission criteria were established: (a) be over the age of 18 and (b) be the father or mother of a child between the ages of 3 and 16 years.

### 2.2 Instruments

This study used a questionnaire ad hoc that collects sociodemographic information on the parents (sex, age, place of residence, educational level, marital status, occupation, and family relationship to the child, i.e., father or mother) and their children (sex, age, occupation, i.e. kindergarten, school, college, high school, professional education or association, disability or nondisability, and type) and the CRI‐A or Inventory of Coping Responses for Adults by Moos (1993) in the version that is adapted and validated on the Spanish sample (Forns et al., 2005). These authors carried out a psychometric analysis of the properties of this scale once translated into Spanish in a sample of 1401 subjects. Regarding internal consistency and reliability, the Cronbach's coefficients alpha reported by Forns et al. (2005) ranged between .40 and .63 of the eight coping strategy scales. The Pearson's correlations between scores on the specific strategy scales vary from .06 to .40. In addition, an exploratory analysis was carried out in which the KMO index and Bartlett's test of sphericity scores showed that the correlation matrix was suitable for factor analysis (KMO = .750; Bartlett: X^2^ = 1658.83, df = 28, *p* < .001). Using the raw scores of the eight specific strategies, a structure matrix produced two factors with eigenvalues greater than 1.00, which together explained 49.6% of variance. Following these analyses, two factors were established (Approach and Avoidance factors), establishing a correlation between the two factors of .35, which indicates that some scales share loadings in both factors. The first factor had a Cronbach's alpha of .68 and the second factor of .55.

Therefore, the CRI‐A inventory consists of two parts. In the first part, participants have to describe the most stressful situation they have experienced in the last 12 months and assess it on a Likert scale based on 10 questions. The second part consists of 48 items with a four‐point scale for a primary assessment of the stressful situation (0 = “never”; 1 = “once or twice”; 2 = “sometimes”; 3 = “often”). According to its authors, the maximum time required for completion is between 10 and 15 min.

The inventory measures avoidance and approach responses to the stressful situation, using eight factors, four factors measure the approach responses and four measure the avoidance responses. To measure the approach responses, the following factors are used: logical analysis (cognitive attempts to understand and mentally prepare to face a stressor and its consequences), positive revaluation (cognitive attempts to build and positively restructure a stressful situation, while accepting the reality of it), seeking support and guidance (behavioral attempts to seek information, support, and guidance), and problem‐solving (behavioral attempts to perform actions directly conducive to the problem). To measure the avoidance responses, the following factors are used: cognitive avoidance (cognitive attempts to avoid thinking about the problem realistically), acceptance or resignation (cognitive attempts to react to the situation by accepting it), search for alternative gratification (behavioral attempts to get involved in alternative activities and create new sources of satisfaction), and emotional discharge (behavioral attempts to reduce tension by expressing negative feelings) (Forns et al., 2005).

The instruments were integrated into a document, accessed online, that the parents had to complete. A parent from each family responded, assessing one problem presented by their youngest child between 3 and 16 years.

### 2.3 Procedure

The selection of the sample was carried out intentionally, in order to have access to the sample with the characteristics described for the group of parents with children with autism spectrum disorder and other disabilities, associations that served and worked directly with these people were selected. Similarly, for the group of parents of children without disabilities included in the sample, regular schools in the same city were selected.

The field study phase involved contacting the directors of educational centers and different associations via telephone to inform them of the investigation. Once approval was obtained, individual appointments were made with the directors of the educational centers and the directors of the associations. The link to the web page containing the sociodemographic questionnaire and CRI‐A was delivered to the centers and associations that subsequently distributed it to families through mailing lists to complete it. The number of potential participants was approximately 400 people; however, the number of actual questionnaires was 140 in total. All participants individually responded to the document through a web link provided in the email, where the reason and need for their collaboration were detailed. All parents were informed of the objectives of the research and accepted the informed consent to participate. It was explained to the parents that the answers, as well as the personal and sociodemographic data, would be treated anonymously and confidentially and that individual data would not be published, but would be used for subsequent statistical analysis as a group.

### 2.4 Design and analysis

A case‐control design with two groups was used (León & Montero, 2015) and one‐way ANOVA with planned contrasts to test for differences between groups was made using the following variables:

- Group variable (GV): parents of children without disabilities (G1), parents of children with ASD (G2), and parents of children with other disabilities (G3).
- Dependent variables (DV): logical analysis (LA), positive revaluation (PR), problem solving (PS), seeking guidance and support (SGS), cognitive avoidance (CA), acceptance or resignation (RA), search for alternative gratification (SAG), and emotional discharge (ED).

To describe reliability, tests were performed to obtain Chronbach's alpha with the results of each scale.

In addition, a chi‐square test was performed to test the possible relationship between the gender of the parents and the disability group to which the children belong, and a one‐factor ANOVA was performed to test possible differences between the age of the parents and the group to which the children belong. Similarly, chi‐square tests have been carried out between the gender of the children and the group to which they belong and a one‐factor ANOVA test for age. On the other hand, homoscedasticity tests of the groups have been carried out to check that the groups were homogeneous and comparable. The statistical analysis package SPSS Version 23 was used to analyze the results.

## 3 RESULTS

Regarding the sample, the total number of participants was 140, although approximately 400 people were contacted, of whom 260 did not answer or did not want to participate in the study.

Before analyzing the results, different tests were carried out to check for potential differences in the sociodemographic variables (gender and age of both parents and children) and the group to which the children belonged, the first of which could be confounding variables.

Regarding the sex of the parents, 75.7% were female (*n* = 106) and 24.3% were male (*n* = 34), with ages ranging from 26 to 64 (*M* = 43.7, *SD* = 6.9). About the sex of the children, 69.3% were male (*n* = 97) and 30.7% (*n* = 43) were female. The children ranged in age from 3 to 16 years old (*M* = 9.39, *SD* = 4.38). Table 1 shows a description of the sociodemographic variables, sex, and age of both parents and children for each of the three groups.

**TABLE 1:** Description of sociodemographic variables (sex and age) of parents and children by group

		(G1) No disabilities			(G2) ASD			(G3) Other disabilities		
		*n*	*M*	SD	*n*	*M*	SD	*n*	*M*	SD
Parents										
Sex	Male	14			15			5		
	Female	56			27			23		
Age			43.93	6.57		44.19	6.40		42.39	8.66
Children										
Sex	Male	37			37			23		
	Female	33			5			5		
Age			9.37	4.32		9.26	4.47		9.61	4.55
*N*		70			42			28		

First, a Pearson's chi‐square test was carried out between the gender of the parents and the group to which the children belong, obtaining nonsignificant values (*X* ^2^ (2) = 4.31, *p* = .12). Similarly, the age of the parents was analyzed according to the group to which the children belonged by means of a one‐factor ANOVA test (*α* = .05), and no significant differences were found between the age of the parents and any of the groups.

As for the variables gender and age of the children and the group to which they belong, a Pearson's chi‐square test was performed between gender and group finding significant data (*X* ^2^(2) = 18.03, *p* = .000). As for the age of the children and the group, no statistically significant differences were found after the one‐factor ANOVA test was performed.

Before carrying out the statistical analysis of all the scales for comparison between groups, a test of homogeneity of variance of the groups was carried out using a Levene's test, finding no statistically significant differences in any group (*p* > .05), so the null hypothesis that the groups are homogeneous is maintained. These results indicate that both groups are statistically equivalent, so it is possible to compare them.

Table 2 below shows the descriptive analyses carried out for all the scales of both factors: approach responses and avoidance responses.

**TABLE 2:** Descriptive analysis of the approach responses and avoidance responses factors and their respective scales of Scores from the CRI‐A

Descriptives					
Factor	Scale		*N*	*M*	SD
Approach responses	LA	No disabilities	70	2.17	0.42
		Autism spectrum disorder	42	1.99	0.46
		Other disabilities	28	2.09	0.45
		Total	140	2.10	0.44
	PR	No disabilities	70	1.76	0.50
		Autism spectrum disorder	42	1.78	0.58
		Other disabilities	28	1.99	0.53
		Total	140	1.81	0.53
	SGS	No disabilities	70	1.73	0.46
		Autism spectrum disorder	42	2.10	0.54
		Other disabilities	28	2.22	0.49
		Total	140	1.94	0.53
	PS	No disabilities	70	2.45	0.42
		Autism spectrum disorder	42	2.45	0.46
		Other disabilities	28	2.47	0.49
		Total	140	2.45	0.44
Avoidance responses	CA	No disabilities	70	1.22	0.73
		Autism spectrum disorder	42	1.40	0.68
		Other disabilities	28	1.56	0.64
		Total	140	1.34	0.71
	AR	No disabilities	70	.85	0.68
		Autism spectrum disorder	42	1.22	0.74
		Other disabilities	28	1.50	0.75
		Total	140	1.09	0.75
	SAG	No disabilities	70	1.33	0.65
		Autism spectrum disorder	42	1.50	0.62
		Other disabilities	28	1.62	0.64
		Total	140	1.44	0.65
	ED	No disabilities	70	1.18	0.60
		Autism spectrum disorder	42	1.42	0.61
		Other disabilities	28	1.54	0.63
		Total	140	1.33	0.62

*N = *number; *M = *mean; SD = standard deviation; LA = logical analysis; PR = positive revaluation; SGS = seeking guidance and support; PS = problem solving; CA = cognitive avoidance; AR = acceptance or resignation; SAG = search for alternative gratification; ED = emotional discharge.

In addition, the analysis performed through a one‐factor ANOVA test with two planned contrasts assuming equal variances (contrast 1: no disability group [G1] versus ASD group and other disabilities group [G2 and G3] and contrast 2: ASD group [G2] versus other disabilities group [G3]) is described.

The data found indicate that there are statistically significant differences (α = .01) in the seeking guidance and support (SGS) scale (*f* (2) = 13.24, *p* = .000), in acceptance or resignation (AR) (*f* (2) = 9.11, *p* = .000), and emotional discharge (ED) (*f* (2) = 4.30, *p* = .015). These data can be seen in Table 3.

**TABLE 3:** One‐factor ANOVA of scores from the CRI‐A

ANOVA							
Factor	Scale	Comparison	Square sum	df	Quadratic mean	*f*	*p*
Approach responses	LA	Between groups	0.87	2	0.43	2.23	.11
		Intragroups	26.77	137	0.19		
		Total	27.63	139			
	PR	Between groups	1.16	2	0.58	2.07	.13
		Intragroups	38.31	137	0.28		
		Total	39.47	139			
	SGS	Between groups	6.45	2	3.22	13.24	.00**
		Intragroups	33.35	137	0.24		
		Total	39.79	139			
	PS	Between groups	0.011	2	0.01	0.03	.97
		Intragroups	27.50	137	0.20		
		Total	27.51	139			
Avoidance responses	CA	Between groups	2.62	2	1.31	2.66	.07
		Intragroups	67.59	137	0.49		
		Total	70.21	139			
	AR	Between groups	9.31	2	4.65	9.11	.00**
		Intragroups	69.98	137	0.51		
		Total	79.29	139			
	SAG	Between groups	1.94	2	0.97	2.36	.10
		Intragroups	56.22	137	0.41		
		Total	58.16	139			
	ED	Between groups	3.19	2	1.59	4.30	.01*
		Intragroups	50.69	137	0.37		
		Total	53.88	139			

df = degrees of freedom; LA = logical analysis; PR = positive revaluation; SGS = seeking guidance and support; PS = problem solving; CA = cognitive avoidance; AR = acceptance or resignation; SAG = search for alternative gratification; ED = emotional discharge.

* *p *< .05.

** *p *< .01.

In reference to the first contrast (G1 vs. G2 plus G3), statistically significant differences are found in the scales of seeking guidance and support (SGS) (*t* (137) = 5.14, *p* = .000) with an effect size (*d *= .79), cognitive avoidance (CA) (*t* (137) = 2. 20, *p* = .029) with an effect size (*d *= .35), acceptance or resignation (AR) (*t* (137) = 4.14, *p* = .000) with an effect size (*d *= .63), search for alternative gratifications (SAG) (*t* (137) = 2.11, *p* = .036) with an effect size (*d *= .34), and emotional discharge (ED) (*t* (137) = 2.91, *p* = .004) with an effect size (*d *= .47). As for the second contrast (G2 vs. G3), no statistically significant differences were found for any of the scales. These data can be seen in Table 4.

**TABLE 4:** One‐factor ANOVA with 2 planned contrasts of scores from the CRI‐A

Factor	Scale	Contrast	Contrast value	SD	*t*	df	*p*
Approach responses	LA	1 (G1–G2G3)	–0.26	0.15	–1.72	137	.09
		2 (G2–G3)	0.10	0.11	0.96	137	.34
	PR	1 (G1–G2G3)	0.26	0.18	1.42	137	.16
		2 (G2–G3)	0.21	0.13	1.64	137	.10
	SGS	1 (G1–G2G3)	0.87	0.17	5.14	137	.00**
		2 (G2–G3)	0.12	0.12	0.97	137	.33
	PS	1 (G1–G2G3)	0.02	0.15	0.15	137	.88
		2 (G2–G3)	0.02	0.11	0.20	137	.84
Avoidance responses	CA	1 (G1–G2G3)	0.53	0.24	2.20	137	.03*
		2 (G2–G3)	0.17	0.17	0.98	137	.32
	AR	1 (G1–G2G3)	1.01	0.24	4.15	137	.00**
		2 (G2–G3)	0.28	0.17	1.59	137	.11
	SAG	1 (G1–G2G3)	0.47	0.22	2.12	137	.04*
		2 (G2–G3)	0.12	0.16	0.80	137	.42
	ED	1 (G1–G2G3)	0.60	0.21	2.91	137	.00**
		2 (G2–G3)	0.12	0.15	0.79	137	.43

SD = standard deviation; df = degrees of freedom; LA = logical analysis; PR = positive revaluation; SGS = seeking guidance and support; PS = problem solving; CA = cognitive avoidance; AR = acceptance or resignation; SAG = search for alternative gratification; ED = emotional discharge.

* *p *< .05.

** *p *< .01.

These analyses indicate that the first null hypothesis of the study (*H0_1_*) should be rejected since there are differences in the coping strategies used by parents of children without disabilities (G1) versus parents of children with disabilities, ASD, and other disabilities (G2 and G3). Therefore, the alternative hypothesis of the study (*H1*) is accepted.

On the other hand, the data seem to indicate that the second null hypothesis of the study (*H0_2_*) should be maintained since there are no significant differences in the coping strategies used by parents of children with ASD (G2) versus other disabilities (G3). Therefore, the second alternative hypothesis of the study (*H2*) is rejected.

Finally, reliability analyses have been carried out for each of the scales, obtaining a moderate or acceptable Cronbach's alpha (*α* = .75).

## 4 DISCUSSION

The data obtained in the present study indicates that there are statistically significant differences in the avoidance responses to the stressful situation. The group of parents of children with disabilities has higher mean scores on the four variables of this dimension, where parents use more cognitive avoidance responses than the parents of children without disabilities. The results show that these parents have higher levels of resignation towards a stressful situation, they tend to look for more satisfying alternatives and emotionally discharge their problems.  However, in the approach responses, significant differences are found in only one of the variables (SGS).

The data seem to indicate that the parents of children with disabilities usually use more avoidance strategies than the other group. Berszan (2017), Cantwell‐Barti (2018), and Twoy et al. (2007) have conducted research in this field, with nonvalidated instruments, small sample size and using only qualitative data. Even with these limitations, they suggest that parents of children with disabilities use avoidance strategies more than parents of children with no disabilities (Berszan, 2017; Cantwell‐Barti, 2018; Twoy et al., 2007). In a quantitative study by Paster et al. (2009), the results are consistent with previous investigations. This study uses ways of coping (Folkman & Lazarus, 1980) to measure the responses of the parents. However, this instrument is criticized for the ambiguity of its items, the lack of and confusing information, variability in the obtained results, and limitations in the factorial analysis (Aldwin & Revenson, 1987; Aliaga & Capafóns, 1996; Folkman et al., 1986; Manne & Zautra, 1989; Parkes, 1984; Scheier et al., 1986; Solomon et al., 1990; Vitaliano et al., 1990).

Following the analysis made in study, it can be pointed out that no significant differences are found in any of the coping responses to the problem, between parents of children with ASD versus other disabilities. No differences were found in approach responses nor avoidance responses. The data indicate that parents of children with disabilities use the same coping strategies, independent of the type of disability that the child has.

The results of this study are surprising; previous investigations have pointed out that families of children with ASD suffer higher levels of stress in comparison to families that are responsible for children with other types of disabilities (Baker et al., 2003; Baker et al., 2005; Kasari & Sigman, 1997; Tomanik et al., 2004; Wolf et al., 1989). The authors point out that to manage these stressors the family have to face them (Berszan, 2017; Brands et al., 2018; Cantwell‐Barti, 2018; Dyson, 2010; Finset & Andersson, 2000; Paster et al., 2009; Tomberg et al., 2005; Twoy et al., 2007). In line with this, we can see, in all the investigations made, avoidance of the problem is the most recurrent response from parents of children with disabilities, both at a cognitive and behavioral level. This type of coping can reduce stress short term but can be detrimental to psychological well‐being (Benson, 2006; Dyson, 2010; Twoy et al., 2007). Thus, different levels of stress and stressors are expected; there would also be different methods or strategies to face the problems caused by them. It all seems to indicate that it is the presence of disabilities and not the type of disability that causes parents to face and experience similar problems. Although, these data should still be taken with caution due to the limited number of participants and ambiguity of the categories created. So far, most studies on coping responses in parents of children with and without disabilities have been made using qualitative methodology, with a relatively small sample size and at times with limited statistical support (see Berszan, 2017; Brands et al., 2018; Cantwell‐Barti, 2018; Finset & Andersson, 2000; Paster et al., 2009; Tomberg et al., 2005; Twoy et al., 2007). Regarding quantitative methodology, only the study by Paster et al. (2009) had been found, using ways of coping as an instrument, which has been widely criticized by several authors (Aldwin & Revenson, 1987; Aliaga & Capafóns, 1996; Folkman et al., 1986; Manne & Zautra, 1989; Parkes, 1984; Scheier et al., 1986; Solomon et al., 1990; Vitaliano et al., 1990).

The present study has been carried out in an attempt to overcome some of the limitations mentioned about previous studies. This investigation supports some methodological aspects and procedures that provide optimal scientific validity: it has the largest sample used so far, it uses a valid instrument (Inventory CRI‐A) that has been adapted and validated in a Spanish sample, it is also the only instrument that measures both cognitive and behavioral responses based on the approach or avoidance of the stressful situation (this instrument had not been used thus far in this field of study), and it is a case‐control design with two groups and presents results supporting inferential statistics.

Until now, no studies have been found that analyze coping responses between parents of children with other types of disabilities, so this study cannot be compared to previous studies. However, an aspect to be taken into account is the reliability values reported (0.4–0.63) by the authors of the Spanish adapted version of the CRI‐A inventory (Forns et al., 2005), which were rather low. A possible limitation of this instrument is the difficulty in the ability to detect differences due to a noisy measure, which should be considered in future research. As a suggestion for future investigations, we recommend using instruments with higher reliability values, and a larger sample of parents of children with other types of disabilities and different categories: for example, intellectual, sensory, or physical disability.

Furthermore, from a methodological point of view, it would be of interest to design studies with larger samples and with a power calculated a priori. In the same way, gender differences between the groups with and without disabilities should be noted as a limitation of the present study so future studies would benefit from controlling for the gender of the children when designing the control groups.

From a theoretical perspective, other variables could be taken into account that could explain possible differences beyond the diagnosis, such as the degree of disability, the presence of disruptive behaviors, social communication problems, participation in parent training programs, among others.

Another possible limitation that should be taken into account for future research is the response bias of the participants. Strategies should be planned to increase the response of potential participants, such as sending out several rounds of questionnaires and personalized letters to encourage greater participation. Additional possible limitations could be that the sample was selected intentional by the characteristics of the sample, due to that, a significant number of parents were found with children that have autism spectrum disorder than of other types of disabilities. Second, it should be noted that the information was collected by a self‐administered questionnaire; this is of a verbal nature and can produce a mismatch between perceived and actual behavior. Based on the results of this study and of previous investigations, future lines of study could analyze the intensity of the stressor and its primary assessment, or appraisal of the problem and its relation to the coping strategies used.

The present investigation has allowed an in‐depth analysis on a field of study that is relatively unexplored, supporting data of great relevance for this collective. This article shows that parents of children with disabilities have less adaptive coping strategies, from a psychological point of view. From this, it can be derived that it is of extraordinary relevance and usefulness to develop prevention and intervention programs that favor adaptive coping strategies.

## 5 CONCLUSION

This research consists of a study with two groups of cases and controls. The data obtained confirmed that parents of children with disabilities were more likely to use avoidance strategies during their most stressful experiences with their child in the last year. These parents scored higher on the dimensions of cognitive avoidance, acceptance or resignation, search for alternative gratification, and emotional discharge and they only use seeking guidance and support as a strategy to approach the problem. However, in the analyses carried out in the sample of parents of children with disabilities (ASD/other disabilities), there are no statistically significant differences in the scores from the questionnaire; thus, they generally use avoidance strategies, regardless of the type of disability that their child has. These data are of special relevance in the field of disability, and they show that the parents of children who present a disability have less adaptive coping strategies in the long term, from a psychological point of view.

## CONFLICT OF INTEREST

The authors declare that they have no conflict of interest.

## AVAILABILITY OF DATA AND MATERIAL

All data collected and subsequently analyzed appear in the manuscript.

## AUTHOR CONTRIBUTIONS

All authors contributed in the same way.

## INFORMED CONSENT

Informed consent was obtained from all individual participants included in the study.

### PEER REVIEW

The peer review history for this article is available at https://publons.com/publon/10.1002/brb3.2701
